# Interference-Limited Absorption in Dense Molecular Nanolayers Near Reflecting Surfaces

**DOI:** 10.1021/acs.jpclett.6c00424

**Published:** 2026-04-14

**Authors:** Zeyu Zhou, Maxim Sukharev, Abraham Nitzan, Joseph E. Subotnik

**Affiliations:** † Department of Chemistry, 6740 Princeton University, Princeton, New Jersey 08544, United States; ‡ Department of Physics, Arizona State University, Tempe, Arizona 85287, United States; ¶ College of Integrative Sciences and Arts, Arizona State University, Mesa, Arizona 85212, United States; § Department of Chemistry, 6572 University of Pennsylvania, 231 South 34th Street, Philadelphia, Pennsylvania 19104, United States; ∥ School of Chemistry, The Raymond and Beverly Sackler Faculty of Exact Sciences and The Sackler Center for computational Molecular and Materials Science, Tel Aviv University, Tel Aviv 6997801, Israel

## Abstract

We investigate linear resonant absorption by a dense ensemble of molecules confined to a sub-wavelength layer in two geometries: (i) a free-standing film in a homogeneous space and (ii) the same film placed at a controlled distance from a reflecting surface. In both cases, increasing the effective light–matter coupling (via molecular density/oscillator strength) produces a nonmonotonic response: absorption rises to an optimum and then decreases as the film becomes increasingly radiatively bright and reflective. Finite-difference time-domain simulations and analytical transfer-matrix calculations agree quantitatively and yield compact ridge conditions for the optimum. We interpret the trends using a scattering/port picture: the isolated film is a symmetric two-port system (reflection and transmission), which bounds single-sided resonant absorption to ≤50% in the ultrathin limit (reflecting transition saturation), whereas adding a mirror suppresses transmission and converts the structure into an effectively one-port absorber. In the mirror-backed geometry, interference can cancel reflection, and unity absorption is obtained at critical coupling, when radiative leakage is balanced by intrinsic molecular loss. These results clarify fundamental limits and design rules for collective absorption in dense molecular layers near dielectric or metallic boundaries.

## Graphical abstract

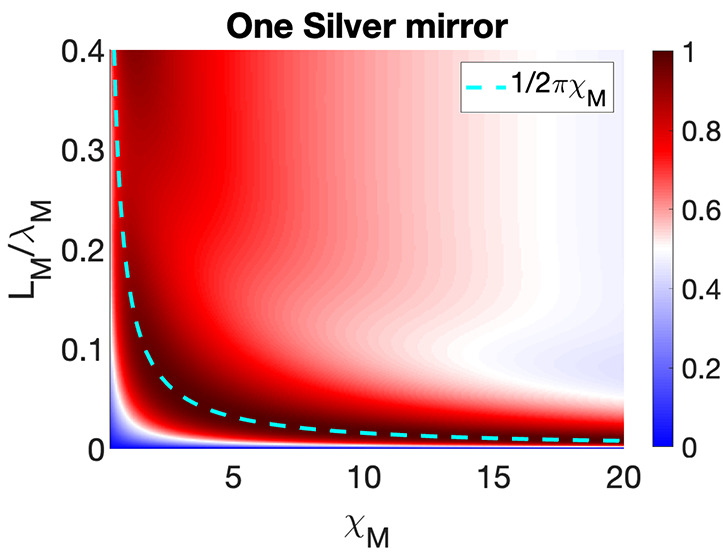

There is today a push to design nanooptical devices with strong optical responses to be used as sensors or for energy harvesting. An important means to control and/or enhance light-matter interactions is to work with molecules located near metallic nanolayers and nanoparticles or dielectric mirrors. One example is surface-enhanced Raman scattering (SERS) where molecular Raman signals can be strongly enhanced near suitable metallic structures.^1–5^ Another is molecular cavity electrodynamics, where hybrid light-matter states are formed inside the nanostructure.^6–16^ Overall, understanding how molecules interact with dielectric inhomogeneous environments remains a fruitful topic of investigation.

To model such a physics, there are two effective approaches. On the one hand, if one can reasonably capture the behavior of a collection of molecules by introducing a dielectric constant that characterizes the molecular environment, then one can use classical dielectric continuum electromagnetic theory to propagate light through just about any material (metallic mirrors, dielectric materials, etc.). For such calculations in simple planar geometries, one can routinely calculate transmission and reflection using a transfer matrix method (TMM).^17^ On the other hand, for nanodevices, if molecule dynamics is important, we must resort to a numerical quantum or semiclassical approach. For the latter, it is standard today to run finite-difference time-domain (FDTD) calculations in the presence of dielectric materials combined with a semiclassical Ehrenfest mean-field description of the matter subsystem. Several recent works have now demonstrated that a host of interesting strong light matter phenomena can in fact be captured via such mixed quantum-classical algorithms.^18–30^ FDTD together with the Ehrenfest approximation is able to describe a host of optical response phenomena of such structures and, when applied to planar interfaces, can capture the physics revealed by the TMM.

In the present paper, we use both TMM and FDTD/Ehrenfest to explore the behavior of a sub-wavelength layer of molecules (or quantum emitters) at high density, where our goal is to analyze how the optical properties of such a layer differ from the properties of a single molecule (emitter). We show that, unlike the case of an isolated molecule, the absorption of such a layer of molecules need not have a monotonic dependence on number density; namely, putting more molecules in the sample need not lead to more absorption. Most importantly, while we analyze such effects in vacuum, we also investigate such optical behavior near a metallic mirror, where the nonmonotonic effect described above is strongly exacerbated. Overall, our results highlight the strong need to study light-matter interactions from a material (rather than molecular) perspective, as several new and unexpected phenomena can be predicted to arise from interference in the high density limit. The central trend reported here, namely, absorption that increases with molecular oscillator strength/density and then decreases, can be understood within the established framework of impedance matching (critical coupling) in planar resonant structures.^31–35^ A subwavelength resonant molecular layer behaves as an effective “absorbing sheet” whose response controls both dissipation and reradiation. In a free-standing (no-mirror) geometry, the layer is a two-port scatterer (reflection and transmission), which imposes a well-known constraint: under single-sided illumination the resonant absorption of a symmetric ultrathin sheet is bounded (approaching 50% in the thin-layer limit).^36,37^ Introducing a reflecting boundary suppresses transmission and turns the system into an effectively one-port absorber; at appropriate layer–mirror separations the reflected field can be canceled by interference, enabling unity absorption when radiative leakage is balanced by material loss, i.e., at the critical coupling strength.^38^ This perspective provides a standard physical interpretation of our ridge conditions and explains why increasing the collective susceptibility beyond an optimum drives the structure into an increasingly reflective regime and reduces the level of absorption.

## Structure of the Systems

In this work, we consider three scenarios probed by a short (1 fs) pulse. In the first and simplest scenario, as shown in Figure 1(a), we place a single layer of 2-level {|*g*⟩, |*e*⟩} systems with energy spacing *E*_*ge*_ = 1.9 eV (wavelength λ_*M*_ = 652 nm) and transition dipole moment μ_*ge*_ = 5 Debye. Without a mirror, we note that all three signals, (i) absorption, (ii) transmission, and (iii) reflection, will be nonzero in general. Second, as shown in Figure 1(b), we place a perfect reflector (black) near the same 2-level molecular layer (red). Here, the perfect reflector does not absorb or allow for the transmission of the EM field. Thus, only (i) molecular absorption (A) and (ii) reflection (R) spectra will be nonzero; they must add to unity (*T* = 0, *R* + *A* = 1). The goal of this scenario is to study the aforementioned interference effects without any nonperfect mirror effects (absorption and phase shift).

**Figure 1:**
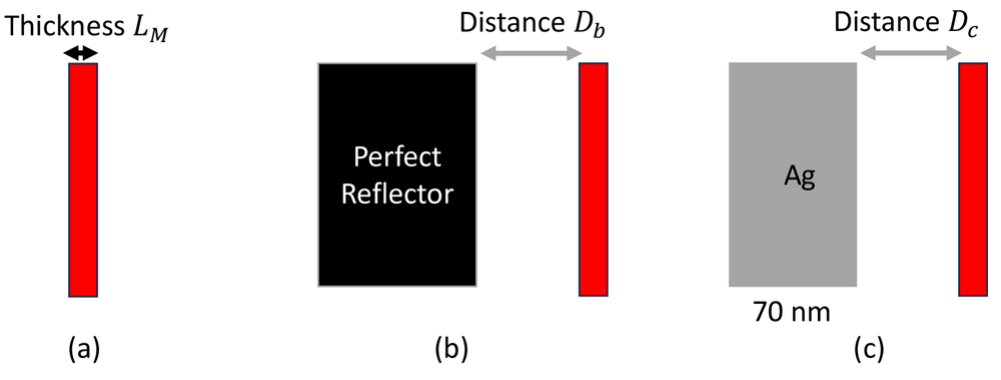
Sketches of the three model scenarios that are studied in this work. (a) A single layer of 2-level molecules with resonant frequency ℏω_*ge*_ = 1.9 eV and thickness *L*_*M*_; (b) a single layer of 2-level molecules with near a perfect reflector; and (c) a single layer of 2-level molecules near a thick silver mirror. Both scenarios are probed by short pulse (1 fs long) from the right-hand side of the system.

Last, as shown in Figure 1(c), we replaced the perfect mirror (black) with a thick (70 nm) silver mirror (gray). This scenario (c) replicates a realistic system that can be experimentally probed. Note that in scenarios (b) and (c), we choose the two distances between the mirror and molecular layers to be different (*D*_*b*_ ≠ *D*_*c*_) for the two different dielectric constants of the mirrors; these distances will be determined below by enforcing maximal absorption.

## Maxwell-Bloch Equations

The interaction between the layers and the external probe is modeled in one-dimension. The one-dimensional Maxwell equations are

1 $$\frac{\partial B_{y}}{\partial t}=-\frac{\partial E_{x}}{\partial z}$$

2 $$\epsilon_{0}\frac{\partial E_{x}}{\partial t}=-\frac{\partial B_{y}}{\mu_{0}\partial z}-J_{x}$$

where, ϵ_0_ is the vacuum permittivity, μ_0_ is the vacuum permeability, *z* is the longitudinal coordinate that is perpendicular to the mirror and molecular slab, and *J*_*x*_ is the polarization current,

3 $$J_{x}=\frac{dP_{x}}{dt}=\varrho \frac{d\left(\mathrm{Tr}\left(\mathrm{\rho ̂}{\mathrm{\mu ̂}}_{x}\right)\right)}{dt}$$

Here, *ϱ* is the number density, and ρ̂ and μ̂_*x*_ are the density matrix and transition dipole operator of the molecular system (see below).

Next, the perfect mirror in scenario (b) is achieved by setting the EM field to 0 at each time step at the boundary. Finally, within the metallic silver mirror, a macroscopic polarization *P*_*x*_ is simulated with the Drude model

4 $$\frac{\partial^{2}P_{x}}{\partial t^{2}}+\gamma \frac{\partial P_{x}}{\partial t}=\epsilon_{0}\Omega_{p}^{2}E_{x}$$

Here, the damping rate γ and plasma frequency Ω_*p*_ are taken to fit the dielectric response of silver at optical frequencies.^39^ Equations 1–4 are discretized in space and time following the finite-difference time-domain (FDTD) approach. For more details regarding the FDTD solver for Maxwell equations with different dielectric constants, please see ref 18. Note that loss in silver is relatively small, and its thickness (70 nm) is large enough so that the results are not much different from those of the perfect mirror.

The molecular layers are described by their 2 × 2 density matrices ρ̂ under a mean-field approximation. In other words, there is one density matrix at each spatial grid point within the molecular layer. The Hamiltonian (and the corresponding Liouvillian) is time-dependent because of the local time-dependent electric field. The system is appended by a phenomenological damping with *T*_1_ = 1 ps to ensure numerical stability. Thus, the equation of motion is

5 $$i\hbar \frac{d}{dt}{\mathrm{\rho ̂}}^{j}=\left[{Ĥ}^{j}\left(t\right),{\mathrm{\rho ̂}}^{j}\right]-\frac{i\hbar }{T_{1}}\left[\begin{array}{cc} -\rho_{ee}^{j} & 1/2\rho_{ge}^{j}\\ 1/2\rho_{eg}^{j} & \rho_{ee}^{j} \end{array}\right]$$

Here, *j* = 1, 2, ..., *N* labels the density matrices at *N* spatial grid points within the molecular layer. The EM field enters the Hamiltonian as the coupling between adjacent electronic states, i.e., $\left\langle g\right|{Ĥ}^{j}\left|e\right\rangle =\mu_{x}^{\mathrm{ge}}E_{x}^{j}\left(t\right)$ :

6 $${Ĥ}^{j}=0\left|g\right\rangle \left\langle g\right|+\hbar \omega_{ge}\left|e\right\rangle \left\langle e\right|+\mu_{x}E_{x}^{j}\left(\left|g\right\rangle \langle e|+\left|e\right\rangle \langle g|\right)$$

All molecular layers are initialized on the ground state (|*g*⟩).^40^ Near the edge of the simulation cell, we drove one grid point for 1 fs as a short pulse. The spatial grid in our simulations is a one-dimensional array from –1000 to 1000 nm, with 4000 grid points. The molecular structure in the three scenarios in Figure 1 is centered at the origin. The laser source is very far from the system (to the right) and generates an EM field traveling in both directions. In the left direction, the generated EM field reaches the molecular layer at normal incidence and *z*_*inc*_ = 950 nm. In the rightward direction, the generated EM field is absorbed by a convolutional perfectly matched layer (CPML) to ensure no reflections as a boundary condition.^18^ For all details of the parameters, please see Appendix C.

At each time step, we record the EM fields on the two sides of the system: transmission and reflection signals. These signals are then Fourier transformed and normalized by the incident amplitudes to obtain the transmission (**T**) and reflection (**R**) spectra (Obviously **T** = 0 for the perfectly reflecting mirror). Finally, the absorption spectra are obtained by **A** = 1 – **T** – **R** and exhibit one single peak at ω = ω_*ge*_ = 1.9 *eV* in all cases. In all figures below, we extract the resonant absorption strength (at ω = ω_*ge*_ = 1.9 eV) and vary other parameters of the systems.

## Dielectric Constant of a 2-Level System

The absorption spectra can also be obtained by the transfer matrix method, provided that we know the correspondence between a two-level system and its complex-valued dielectric constant. Using the Lorentz model described in ref 24, the dielectric constant is written in the form:

7 $$\chi_{e}\left(\omega \right)=\frac{2\mu_{x}^{2}\varrho \omega_{ge}}{\varepsilon_{0}\left(\omega_{ge}^{2}-\omega^{2}+i\omega \Gamma \right)}$$

8 $$n\left(\omega \right)=\sqrt{1+\chi_{e}\left(\omega \right)}$$

Here, ℏΓ = 2πℏ/*T*_1_ ≈ 4 × 10^–3^ *eV* represents the phenomenological damping.

We focus on the resonant condition in which the electric susceptibility is purely imaginary:

9 $$\chi_{e}\left(\omega =\omega_{ge}\right)=-\frac{2i\mu_{x}^{2}\varrho }{\varepsilon_{0}\Gamma }$$

Below, we define χ_*M*_ = |χ_*e*_(ω = ω_*ge*_)| for characterizing the effective light-matter interaction. It is determined by the transition dipole moment μ, the number density *ϱ*, and the lifetime (1/Γ). Note that 1 + χ_*e*_(ω) is formally complex-valued; when calculating the dielectric constant *n* in eq 8, we choose the square root with negative imaginary part (I(n)≤0) meaning the molecular layer will be absorptive.

All results in Figure 2 are obtained by FDTD calculations and are validated with the transfer matrix method. For detailed discussions of the transfer matrix method, see Appendix A. Let us now begin by investigating how the distance between the mirror and the molecular layer changes the absorption strength. We also vary the effective electric susceptibility at resonance to connect the absorption strength and physical properties of the molecular layer such as number density and transition dipole moment strength. Thereafter, we study the absorption as a function of varying molecular layer thickness and single molecule electric susceptibility, and we present an analytical formula (see below eq 10) for the absorption maxima.

## Absorption Intensity at Varying Mirror-Molecular Layer Distance

To study the dependence of the absorption on the distance *D* we use a thin (*L*_*M*_ = 10 nm ≪ *D*) molecular layer and calculate the absorption as *D* changes within one wavelength. Figure 2(a) shows the absorpion calculated using the TMM (red lines) and FDTD (blue lines). Note that both methods are in agreement here, and the overall prediction is that one finds a peak near *D* = $\left(2N+1\right)\lambda_{M}/4$ where N∈N . At this distance, the reflected light undergoes maximal constructive interference with the incident light: for a perfect mirror, this distance arises because during reflection, the mirror provides an extra π phase shift (and thus an effective λ_*M*_/2 light path) which, when combined with another λ_*M*_/2 light path for the round trip between the molecular layer and the mirror, yields a constructive λ_*M*_ change in phase. From Figure 2, we extract the optimal distances (the fundamental interference distance with maximal absorption) *D* between the molecular layer and the perfect mirror *D*_*b*_ ≈ 163 nm. In Appendix B, we have also conducted a similar simulation for a silver mirror, for which we obtain *D*_*c*_ ≈ 135 nm. These distances are fixed below.

**Figure 2:**
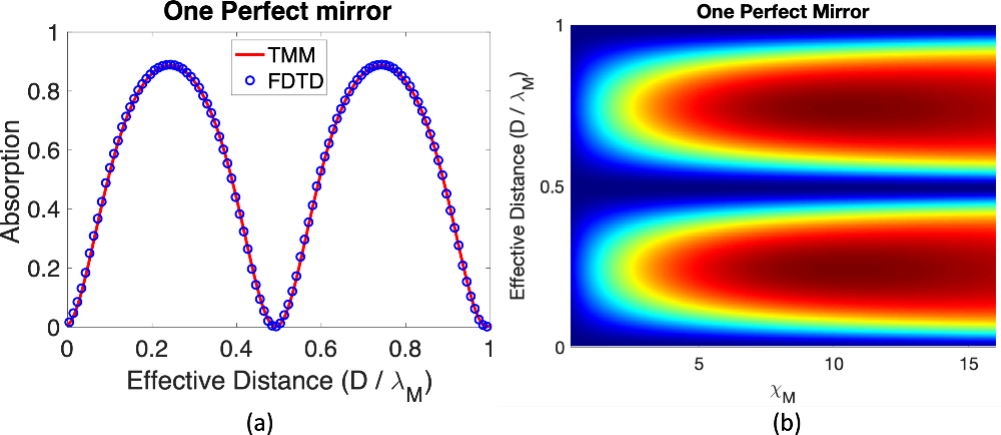
(a) Absorption intensity at ℏω = 1.9 eV for varying the mirror-molecular layer distance. The thickness of the molecular layer is *L*_*M*_ = 10 nm, the number density is 1 mol/L, the transition dipole moment is 5 Debye, and these parameters correspond to molecular susceptibility χ_*M*_ = 5.16. The results are probed by a very weak short pulse and excite minimal population onto the excited state throughout the simulation. (b) Absorption intensity plotted against the mirror-molecular layer distance *D* and the molecular susceptibility χ_*M*_ for a perfect reflector. The perfect mirror gives a clear pattern: the maximal absorption values appear at D= $\left(2N+1\right)\lambda_{M}/4$ where N∈N and minimal absorption appears at D= $N\lambda_{M}/2$ where N∈N . This pattern arises due to the constructive/destructive interference of the standing wave.

Another aspect of the absorption behavior is seen in Figure 2(b), where we make a heatplot of the absorption as a function of both the distance *D* and the molecular layer susceptibility χ_*M*_. The oscillations with distance *D* reflect the behavior seen in Figure 2(a). In addition, we observe that the absorption intensity reaches a maximum (**A**(ω = ω_*ge*_) = 1, **R** + **T** = 0) at χ_*M*_ ≈ 10.5. Note that the calculation is conducted with a short input white light pulse. Finally we note that while considering absorption is a standard way to study linear optical response, in the present context transmission and reflection are the natural accessible observables. The corresponding reflection peaks (see Appendix A, eqs 26 and 39) show the same physical behavior as in Figure 2.

## Resonant Absorption Intensity for Varying Molecular Layer Thickness

The data above leave a puzzling question: why is there an absorption peak as a function of χ_*M*_, the light-matter coupling? How does this result intimately tied to the existence of the mirror? To best answer the questions above, we will next probe the absorption intensity with no mirror nearby. We allow the molecular layer thickness to change as a free parameter (*L*_*M*_) on the vertical axis in Figure 3. All data from Figure 3 are generated from a TMM method.

**Figure 3:**
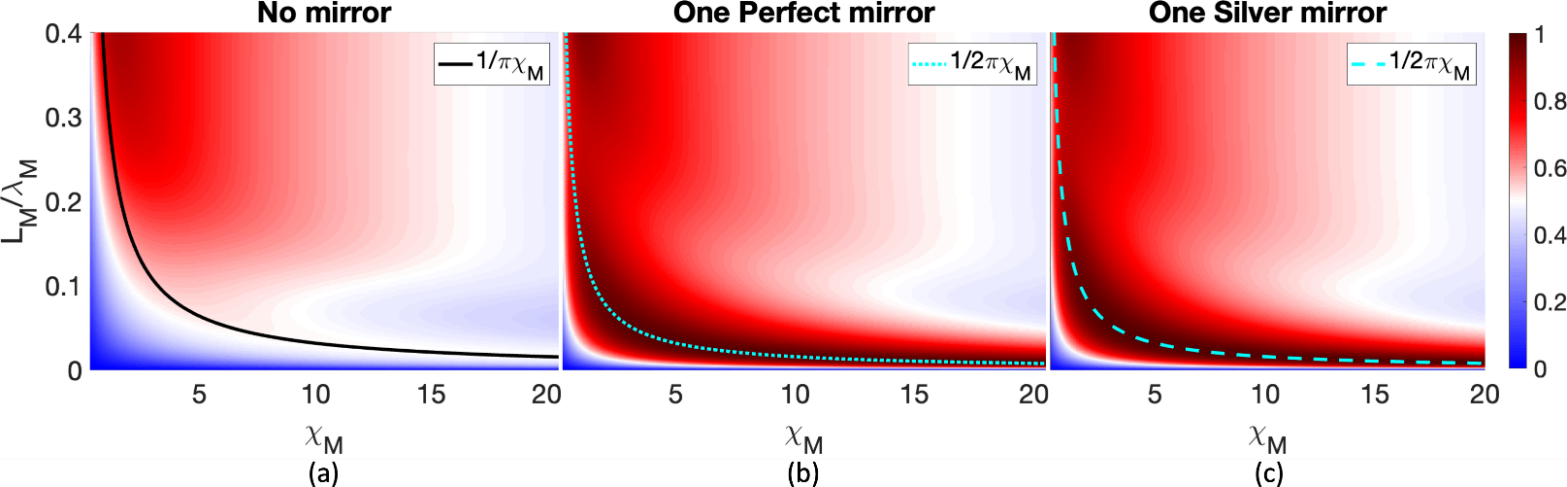
Maximal absorption at ℏω = ℏω_*ge*_ = 1.9 eV for varying molecular layer thickness and electric susceptibility. (a) No mirror, (b) one perfect mirror at distance (*D* = 163 nm), and (c) one 70 nm silver mirror at distance (*D* = 135 nm). In (a), because the transmission channel is also finite in scenario (a) and thus by symmetry, the boundary is defined as *A* = 0.5 (white band) and is at *L*_*M*_/λ_*M*_ = 1/*πχ*_*M*_. In contrast, as shown in parts b and (c), the maximal absorption (*A* = 1, dark red band) is reached at *L*_*M*_/λ_*M*_ = 1/2*πχ*_*M*_ (cyan lines). In all figures, the lines are most accurate in the limit of *L*_*M*_/λ_*M*_ < 0.05.

According to Figure 3(a), we find two regimes in the resonant absorption intensity: *L*_*M*_ < 0.05λ_*M*_ vs *L*_*M*_ ≥ 0.05λ_*M*_. As shown in Figure 3 (a), in the limit of a thin layer thickness *L*_*M*_ < 0.05λ_*M*_ for the no mirror scenario (a), we still find a maximum in absorption, but now at *A* = 0.5 (white band, highlighted by the black solid line); although perhaps faint to the eye, the absorption on both sides of the ridge is smaller than a half, *A* ≤ 0.5 (blue areas). Interestingly, several of these features change in the opposite limit of the large thickness (*L*_*M*_). Here, as one might normally expect, we can find a scenario where *A* = 1 (full absorption). Such a peak (dark red) occurs at χ_*M*_ ≈ 2; for larger values of χ_*M*_, the absorption intensity is reduced (presumably as the molecular layer acts more like a mirror).

The data in Figure 3(a) are quite illuminating, insofar as they offer new insight into the different regimes possible. To that end, we next extract the observables for scenarios (b) and (c), as shown in Figure 3 panels (b) and (c). Now, in the limit of thin layer thickness *L*_*M*_ < 0.05λ_*M*_, the maximal absorption is at *A* = 1 (dark red band, highlighted by cyan dotted and dashed lines) rather than *A* = 0.5. As already demonstrated in Figure 2, the nearby mirror enhances the resonant absorption, and thus the blue (low absorption) regions near the ridge are narrower than those in the no mirror scenario (a). Finally, as the thickness of the molecular layer *L*_*M*_ increases, the resonant absorption is overall stronger than in the scenario without a mirror, while the absorption pattern is similar.

Overall, in comparing Figure 3 panel (a) with panels (b) and (c), the major differences are in the limit *L*_*M*_ ≪ λ_*M*_, where the absorption peak is larger (*A* = 1 vs *A* = 0.5) and stronger with a mirror as compared to without a mirror. To better understand these effects, we initiated a series of TMM calculations. As shown in Appendix A, one can analytically show that indeed, for a narrow molecular layer, the maximal absorption condition becomes χ_*M*_ = *k*_*M*_ *L*_*M*_ which yields *A* = 1 in the presence of a mirror. From eq 9, this expression can be written as follows.

10 $$\varrho \mu_{x}^{2}=\frac{\varepsilon_{0}\pi \Gamma L_{M}}{\lambda_{M}}$$

Without a mirror, the corresponding condition becomes χ_*M*_ = *k*_*M*_ *L*_*M*_/2 which yields *A* = 0.5.

The fact that the results above can be indeed recovered by a simple TMM calculation (with a simple form for the optimal number density in terms of lengths and wavelengths) indicates that there must be a reasonable, simple, intuitive explanation. In short, it would appear that for a single molecular layer, a majority of light will pass through the molecular layer once and leave. Therefore, as the molecular susceptibility increases, the reflection signal increases from 0 to 1 while the transmission signal decreases from 1 to 0. By symmetry, one would expect the maximal absorption to be half (i.e., the other half stays in the form of light and moves away). In contrast, for a molecule near a mirror, the transmission is completely blocked, and light can pass through the molecular layer more than once. Hence, the light can move back and forth between the mirror and molecular layer, and a larger number of wavefronts would seem to allow for more complete interference and stronger maxima. In any event, one big conclusion is that we need to be careful with using single molecular analogy when considering a subwavelength molecular nanolayer because this nanolayer can exhibit unusual collective responses.

To best reinforce this caveat, in Figure 4, we plot the absorption on resonance for a two-level system as a function of the number density, *ϱ* (see eq 9). Note that there is a maximum, highlighting that higher molecular density does not always result in higher absorption. Alternatively, if we focus on the low number density limit, the curve clearly violates Lambert’s law: as the number density increases from 0, the absorption gradually deviates from a linear relation. However, note that even as we increase the number density, every two-level system remains barely excited and very far from saturated; again the excitation here is collective and cannot be understood in terms of single molecules.

**Figure 4:**
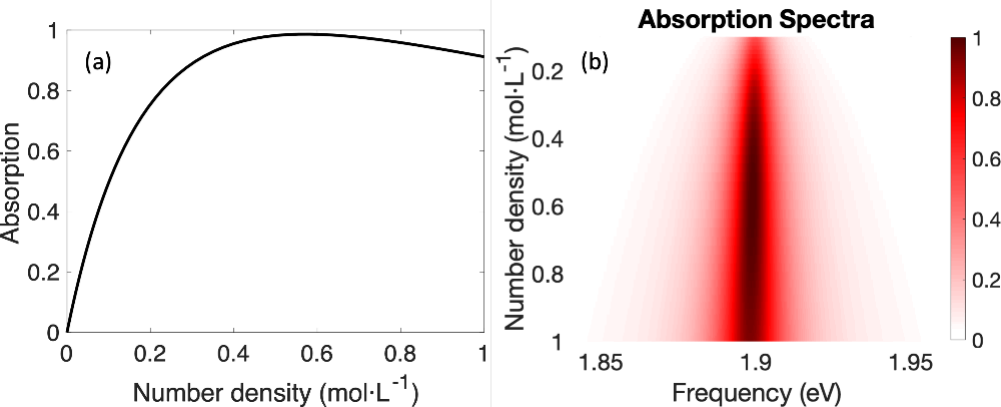
(a) Nonmonotonic absorption intensity as a function of the number density at incoming frequency 1.9eV. (b) Heatmap of the absorption as a function of both number density and incoming frequency. The nonobvious peak in absorption as a function of number density can be explained analytically by a TMM calculation in Appendix A.

Our analytic conditions can be interpreted as explicit matching conditions between radiative and dissipative channels. In the no-mirror case, the molecular nanolayer necessarily partitions incident power among reflection, transmission, and absorption; increasing the collective susceptibility strengthens both absorption and coherent reradiation, and beyond a certain point the enhanced back scattering prevents efficient energy delivery into the layer. Consequently, the absorption exhibits a maximum consistent with the thin film two-port bound (approaching *A*_*max*_ ≈ 0.5 for a symmetric ultrathin resonant sheet under single sided excitation) rather than growing monotonically with oscillator strength or density.

With a mirror present, transmission is eliminated, and absorption is controlled primarily by the cancellation (or enhancement) of reflection. At mirror-layer separations that place the molecular sheet near a field antinode (approximately a quarterwave, modified by the mirror reflection phase), the structure behaves as a mirror-backed absorbing sheet analogous to a Salisbury-screen-type absorber. In this one-port configuration, the derived optimal susceptibility corresponds to critical coupling: radiative leakage into the single external channel is matched by internal molecular loss, producing a reflection minimum and (at the optimum) a perfect absorption. When the collective susceptibility exceeds this point, the system becomes overcoupled/over-reflective and absorption decreases (providing a unified explanation for the observed nonmonotonic dependence of resonant absorption on coupling strength).

In summary, we investigated the enhancement of resonant absorption spectra of a subwavelength molecular nanolayer by a nearby metallic surface. The resonant absorption intensities for varying mirror-molecular layer distance, molecular layer thickness, and effective light-matter interactions are obtained by transfer matrix method (TMM) and finite difference time domain (FDTD) calculations. We have found that the presence of a mirror enhances the absorption intensity in three aspects: (i) Distance-dependent absorption affected by interference between incoming and reflected light with absorption maxima at distances approximately (2*N* + 1)­λ_*M*_/4, where constructive interferences maximize the local EM field; (ii) there is an optimal pair of light-matter interaction and thickness for maximizing absorption that is different from a bare molecular layer; and (iii) a mirror can make the maximal point both more pronounced (a full absorption) and easier to reach (less collective molecular susceptibility χ_*M*_). Most importantly, for a given choice of molecular layer thickness, as we increase the effective light-matter coupling, we observe stronger absorption until a maximum point is reached. However, if we further increase the light-matter coupling, the absorption intensity decreases (which must be due to a convoluted phase alignment between multiple wavefronts) according to both simulation and analytic considerations (see Appendix A). The root cause is a complex interference effect of many wavefronts; effectively, a molecular layer with its own dielectric must itself act like a cavity to some extent, which automatically leads to a maximum condition for absorption determined by interference; when a mirror is present, that condition is further enhanced.

Looking forward, for a properly chosen set of target molecules, we believe the effect above should be measurable, where we predict a violation of Lambert’s law and an enhancement effect due to a mirror. (Of course, a quantitative interpretation will require properly accounting for intermolecular coupling^28^ when treating a real material.) The resulting analytic “ridge” conditions provide practical design rules for tuning between reflection dominated, transparency-like, and perfect-absorption regimes in dense resonant films, offering a compact route to engineer narrowband spectral features and strong field–matter interaction in planar nanophotonic architectures. Finally, this work highlights that collective responses from a dense molecular layer can be drastically different from standard intuitions based on a single-molecular layer. In particular, in the nanoregime—with dense molecular layers situated between molecular and bulk length scales—the resonance condition in eq 10 offers us an important guide to designing materials and nanodevices with unique responses and high sensitivity.

## Figures and Tables

**Figure 5:**
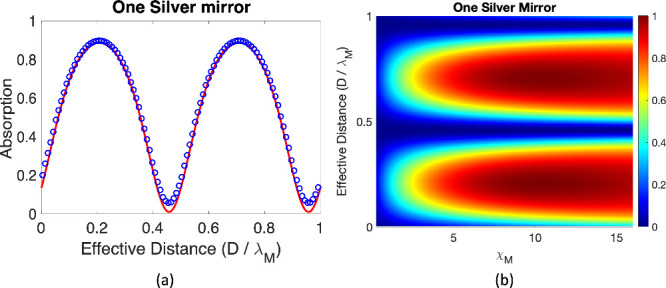
Absorption at resonance, ℏω = ℏω_*ge*_ = 1.9 eV plotted against (a) the mirror-molecular layer distance *D* and (b) the molecular susceptibility χ_*M*_ for a silver mirror. The silver mirror possesses a finite complex-valued dielectric constant, and thus the dependence of maximal/minimal absorption on effective distance deviates from Figure 2(a) at around *D* = 0.45λ_*M*_. The thickness of molecular layer is 10 nm.

## Appendix A: Deriving the Maximum Boundaries with Transfer Matrix Method

As shown in Figure 3, the two maximal absorption boundaries are critical to understand the full absorption diagram. Thus, in this appendix, we analytically derive these boundaries with the transfer matrix method (TMM). We will start by introducing the transfer matrix method.

For layered systems, the central physical quantity is the real or complex-valued dielectric constant (*n*). Thereby, for normal incidence onto a layer with thickness *L*, we define matrices:

11
$$D=\left[\begin{array}{cc} 1 & 1\\ n & -n \end{array}\right]$$

12
$$D^{-1}=\frac{\left[\begin{array}{cc} 1 & 1/n\\ 1 & -1/n \end{array}\right]}{2}$$

13
$$P\left(L\right)=\left[\begin{array}{cc} \exp\left(inkL\right) & 0\\ 0 & \exp\left(-inkL\right) \end{array}\right]$$

Here, **D** describes the interface and **P** describes the propagation inside layer. *k* is the wavevector of the incident light. The full transfer matrix **M** across such a layer is

14
$$M=\mathrm{DP}\left(L\right)D^{-1}=\frac{\left[\begin{array}{cc} \exp\left(inkL\right)+\exp\left(-inkL\right) & \left(\exp\left(inkL\right)-\exp\left(-inkL\right)\right)/n\\ n\left(\exp\left(inkL\right)-\exp\left(-inkL\right)\right) & \exp\left(inkL\right)+\exp\left(-inkL\right) \end{array}\right]}{2}$$

Here, **M** carries information on how the phase of the EM field with wavevector *k* evolves after passing through the whole layer, and we can extract transmission and reflection from **M**. For more details of TMM, there is a comprehensive discussion in ref 17. Note that we do not reduce the matrix elements to trigonometric functions because, in general, the dielectric constant *n* is complex-valued. Below, we will separately derive the two boundary lines: No mirror (Figure 3(a)) vs One mirror (Figure 3(b)).

### A.1 No Mirror

The full transfer matrix for any dielectric layer with thickness *L* is

M=Dvac−1DP(L)D−1Dvac(15)=[111−1][exp(inkL)+exp(−inkL)(exp(inkL)−exp(−inkL))/nn(exp(inkL)−exp(−inkL))exp(inkL)+exp(−inkL)][111−1]4(16)

Here, we focus on the first column of the matrix (**M**_11_ and **M**_21_):

17
$$M_{11}=\frac{\left(n+2+1/n\right)\exp\left(inkL\right)+\left(2-n-1/n\right)\exp\left(-inkL\right)}{4}$$

18
$$M_{21}=\frac{\left(1/n-n\right)\exp\left(inkL\right)+\left(n-1/n\right)\exp\left(-inkL\right)}{4}$$

Next, we calculate the absolute square of the two matrix elements:

19
$$16{\left|M_{11}\right|}^{2}={\left|n+2+1/n\right|}^{2}\exp\left(-2I\left(n\right)kL\right)+{\left|2-n-1/n\right|}^{2}\exp\left(2I\left(n\right)kL\right)+\left(n+2+1/n\right)\left(2-n^{*}-1/n^{*}\right)\exp\left(2iR\left(n\right)kL\right)+\left(n^{*}+2+1/n^{*}\right)\left(2-n-1/n\right)\exp\left(-2iR\left(n\right)kL\right)$$

20
$$8{\left|M_{11}\right|}^{2}=\left(4+{\left|n\right|}^{2}+\frac{1+2R{\left(n\right)}^{2}-2I{\left(n\right)}^{2}}{{\left|n\right|}^{2}}\right)\cosh\left(2I\left(n\right)kL\right)-\left(4R\left(n\right)+\frac{4R\left(n\right)}{{\left|n\right|}^{2}}\right)\sinh\left(2I\left(n\right)kL\right)+\left(4-{\left|n\right|}^{2}-\frac{1+2R{\left(n\right)}^{2}-2I{\left(n\right)}^{2}}{{\left|n\right|}^{2}}\right)\cos\left(2R\left(n\right)kL\right)+\left(4I\left(n\right)-\frac{4I\left(n\right)}{{\left|n\right|}^{2}}\right)\sin\left(2R\left(n\right)kL\right)$$

21
$$8{\left|M_{21}\right|}^{2}={\left|n-\frac{1}{n}\right|}^{2}\left(\cosh\left(2I\left(n\right)kL\right)-\cos\left(2R\left(n\right)kL\right)\right)=\left({\left|n\right|}^{2}+\frac{1-2R{\left(n\right)}^{2}+2I{\left(n\right)}^{2}}{{\left|n\right|}^{2}}\right)\times \left(\cosh\left(2I\left(n\right)kL\right)-\cos\left(2R\left(n\right)kL\right)\right)$$

Here, R(n) is the real part and I(n) is the imaginary part of the dielectric constant *n*, and thus ${\left|n\right|}^{2}=R{\left(n\right)}^{2}+I{\left(n\right)}^{2}$ . As shown in Figure 3, we focus on dense molecules/strong transition dipole moment regime. Thus, the boundary line is most accurate for small thickness *L* and large electric susceptibility *χ*_*M*_ regime (lower right). In this limit, we make the following approximation:

22
$$\begin{array}{l} n_{M}=\sqrt{1+\chi_{e}}\approx \sqrt{-i\chi_{M}}\equiv \left(1-i\right)x\\ x\approx R\left(n\right)\approx -I\left(n\right)\geq 0 \end{array}$$

23
2kL≡a≥0

Thus, the two matrix elements in eqs 20 and 21 become

24
$${\left|M_{11}\right|}^{2}\approx \left(\frac{8x^{4}+4x^{2}+1}{16x^{2}}\right)\cosh\left(ax\right)+\left(\frac{2x^{2}+1}{4x}\right)\sinh\left(ax\right)+\left(\frac{8x^{4}-4x^{2}-1}{16x^{2}}\right)\cos\left(ax\right)+\left(\frac{2x^{2}-1}{4x}\right)\sin\left(ax\right)\approx \frac{8x^{4}+4x^{2}+1}{16x^{2}}\left(1+\frac{a^{2}x^{2}}{2}\right)+\frac{2ax^{2}+a}{4x}+\left(\frac{8x^{4}-4x^{2}-1}{16x^{2}}\right)\left(1-\frac{a^{2}x^{2}}{2}\right)+\frac{2ax^{2}-a}{4}=\left(\frac{ax^{2}}{2}+1\right)+\frac{a^{2}}{16}$$

25
$$|M_{21}{|}^{2}\approx \left(\frac{x^{2}}{4}+\frac{1}{16x^{2}}\right)\left(\cosh\left(ax\right)-\cos\left(ax\right)\right)\approx \left(\frac{a^{2}x^{4}}{4}+\frac{a^{2}}{16}\right)$$

The final absorption is obtained by

26
$$A=1-T-R=\frac{\left|M_{11}{|}^{2}-1-\right|M_{21}{|}^{2}}{|M_{11}{|}^{2}}=\frac{4ax^{2}}{{\left(ax^{2}+2\right)}^{2}+\frac{a^{2}}{4}}\equiv \frac{4x^{\prime }}{{\left(x^{\prime }+2\right)}^{2}+\frac{a^{2}}{4}}$$

Here, *x*′ = *ax* ^2^. Let us calculate the derivative with respect to *x*′ and find the maximum:

∂A∂x′=a2−4x′2+16((x′+2)2+a24)2=0(27)⇒2πLMχM/λM=ax2=x′=4+a2/4≈2(28)

Thus, we obtain an approximate analytical formula for the ridge in the limit of small thickness for Figure 3(a): *π L*_*M*_ *χ*_*M*_/*λ*_*M*_ = 1. The maximal value is

29
$$A\left(x^{\prime }\approx 2\right)=\frac{8}{16+\frac{a^{2}}{4}}\approx \frac{1}{2}$$

Note that this approximate formula breaks down in the limit of large thickness (*L*_*M*_/*λ*_*M*_ > 0.05), as shown in Figure 3.

### A.2 One Perfect Mirror

The transfer matrix D(M) for a perfect mirror is

30
$$D\left(M\right)=\left[\begin{array}{cc} 1 & 1\\ M & -M \end{array}\right]$$

Here, for a perfect mirror, M is a big number so that below we will omit all terms without M . The full transfer matrix for the system in Figure 1(b) is

31
$$M^{\prime }=D_{vac}^{-1}\mathrm{DP}\left(L\right)D^{-1}D_{vac}P_{vac}\left(D\right)D_{vac}^{-1}D\left(M\right)=\frac{\left[\begin{array}{cc} 1 & 1\\ 1 & -1 \end{array}\right]\left[\begin{array}{cc} \exp\left(inkL\right)+\exp\left(-inkL\right) & \left(\exp\left(inkL\right)-\exp\left(-inkL\right)\right)/n\\ n\left(\exp\left(inkL\right)-\exp\left(-inkL\right)\right) & \exp\left(inkL\right)+\exp\left(-inkL\right) \end{array}\right]\left[\begin{array}{cc} \cos\left(kD\right) & i\sin\left(kD\right)\\ i\sin\left(kD\right) & \cos\left(kD\right) \end{array}\right]\left[\begin{array}{cc} 1 & 1\\ M & -M \end{array}\right]}{2}\approx \frac{\left[\begin{array}{cc} 1 & 1\\ 1 & -1 \end{array}\right]\left[\begin{array}{cc} \exp\left(inkL\right)+\exp\left(-inkL\right) & \left(\exp\left(inkL\right)-\exp\left(-inkL\right)\right)/n\\ n\left(\exp\left(inkL\right)-\exp\left(-inkL\right)\right) & \exp\left(inkL\right)+\exp\left(-inkL\right) \end{array}\right]\left[\begin{array}{cc} iM\sin\left(kD\right) & -iM\sin\left(kD\right)\\ M\cos\left(kD\right) & -M\cos\left(kD\right) \end{array}\right]}{2}$$

Again, we focus on the two elements of the first column:

32
$$M_{11}^{\prime }=\frac{M}{2}\left\{\left(n+1\right)\exp\left(inkL\right)\left(i\sin\left(kD\right)+\frac{\cos\left(kD\right)}{n}\right)+\left(1-n\right)\exp\left(-inkL\right)\left(i\sin\left(kD\right)-\frac{\cos\left(kD\right)}{n}\right)\right\}$$

33
$$M_{21}^{\prime }=\frac{M}{2}\left\{\left(1-n\right)\exp\left(inkL\right)\left(i\sin\left(kD\right)+\frac{\cos\left(kD\right)}{n}\right)+\left(n+1\right)\exp\left(-inkL\right)\left(i\sin\left(kD\right)-\frac{\cos\left(kD\right)}{n}\right)\right\}$$

Next, we take the absolute square of the two elements:

34
$$\frac{4{\left|M_{11}^{\prime }\right|}^{2}}{M^{2}}={\left|1+n\right|}^{2}\left(\sin^{2}\left(kD\right)+\frac{\cos^{2}\left(kD\right)+2I\left(n\right)\sin\left(kD\right)\cos\left(kD\right)}{{\left|n\right|}^{2}}\right)\exp\left(-2I\left(n\right)kL\right)+{\left|1-n\right|}^{2}\left(\sin^{2}\left(kD\right)+\frac{\cos^{2}\left(kD\right)-2I\left(n\right)\sin\left(kD\right)\cos\left(kD\right)}{{\left|n\right|}^{2}}\right)\exp\left(2I\left(n\right)kL\right)+\left(1-{\left|n\right|}^{2}+2iI\left(n\right)\right)\left(\sin^{2}\left(kD\right)-\frac{\cos^{2}\left(kD\right)-2iR\left(n\right)\sin\left(kD\right)\cos\left(kD\right)}{{\left|n\right|}^{2}}\right)\exp\left(2iR\left(n\right)kL\right)+\left(1-{\left|n\right|}^{2}-2iI\left(n\right)\right)\left(\sin^{2}\left(kD\right)-\frac{\cos^{2}\left(kD\right)+2iR\left(n\right)\sin\left(kD\right)\cos\left(kD\right)}{{\left|n\right|}^{2}}\right)\exp\left(-2iR\left(n\right)kL\right)$$

35
$$\frac{4{\left|M_{21}^{\prime }\right|}^{2}}{M^{2}}={\left|1-n\right|}^{2}\left(\sin^{2}\left(kD\right)+\frac{\cos^{2}\left(kD\right)+2I\left(n\right)\sin\left(kD\right)\cos\left(kD\right)}{{\left|n\right|}^{2}}\right)\exp\left(-2I\left(n\right)kL\right)+{\left|1+n\right|}^{2}\left(\sin^{2}\left(kD\right)+\frac{\cos^{2}\left(kD\right)-2I\left(n\right)\sin\left(kD\right)\cos\left(kD\right)}{|n{|}^{2}}\right)\exp\left(2I\left(n\right)kL\right)+\left(1-{\left|n\right|}^{2}-2iI\left(n\right)\right)\left(\sin^{2}\left(kD\right)-\frac{\cos^{2}\left(kD\right)-2iR\left(n\right)\sin\left(kD\right)\cos\left(kD\right)}{{\left|n\right|}^{2}}\right)\exp\left(2iR\left(n\right)kL\right)+\left(1-{\left|n\right|}^{2}+2iI\left(n\right)\right)\left(\sin^{2}\left(kD\right)-\frac{\cos^{2}\left(kD\right)+2iR\left(n\right)\sin\left(kD\right)\cos\left(kD\right)}{{\left|n\right|}^{2}}\right)\exp\left(-2iR\left(n\right)kL\right)$$

We make the same approximation as eqs 22 and 23:

36
$$\frac{4{\left|M_{11}^{\prime }\right|}^{2}}{M^{2}}\approx \left(1+2x^{2}+2x\right)\left(\sin^{2}\left(kD\right)+\frac{\cos^{2}\left(kD\right)-2x\sin\left(kD\right)\cos\left(kD\right)}{2x^{2}}\right)\exp\left(ax\right)+\left(1+2x^{2}-2x\right)\left(\sin^{2}\left(kD\right)+\frac{\cos^{2}\left(kD\right)+2x\sin\left(kD\right)\cos\left(kD\right)}{2x^{2}}\right)\exp\left(-ax\right)+\left(1-2x^{2}-2ix\right)\left(\sin^{2}\left(kD\right)-\frac{\cos^{2}\left(kD\right)-2\mathrm{ix}\sin\left(kD\right)\cos\left(kD\right)}{2x^{2}}\right)\exp\left(iax\right)+\left(1-2x^{2}+2ix\right)\left(\sin^{2}\left(kD\right)-\frac{\cos^{2}\left(kD\right)+2\mathrm{ix}\sin\left(kD\right)\cos\left(kD\right)}{2x^{2}}\right)\exp\left(-iax\right)=\left\{2\left(1+2x^{2}\right)\left(\sin^{2}\left(kD\right)+\frac{\cos^{2}\left(kD\right)}{2x^{2}}\right)-4\sin\left(kD\right)\cos\left(kD\right)\right\}\cosh\left(ax\right)+\left\{4x\left(\sin^{2}\left(kD\right)+\frac{\cos^{2}\left(kD\right)}{2x^{2}}\right)-\frac{2\left(1+2x^{2}\right)}{x}\sin\left(kD\right)\cos\left(kD\right)\right\}\sinh\left(ax\right)+\left\{2\left(1-2x^{2}\right)\left(\sin^{2}\left(kD\right)-\frac{\cos^{2}\left(kD\right)}{2x^{2}}\right)+4\sin\left(kD\right)\cos\left(kD\right)\right\}\cos\left(ax\right)+\left\{4x\left(\sin^{2}\left(kD\right)-\frac{\cos^{2}\left(kD\right)}{2x^{2}}\right)-\frac{2\left(1-2x^{2}\right)}{x}\sin\left(kD\right)\cos\left(kD\right)\right\}\sin\left(ax\right)\approx \left\{2\left(1+2x^{2}\right)\left(\sin^{2}\left(kD\right)+\frac{\cos^{2}\left(kD\right)}{2x^{2}}\right)-4\sin\left(kD\right)\cos\left(kD\right)\right\}\left(1+a^{2}x^{2}/2\right)+\left\{4x\left(\sin^{2}\left(kD\right)+\frac{\cos^{2}\left(kD\right)}{2x^{2}}\right)-\frac{2\left(1+2x^{2}\right)}{x}\sin\left(kD\right)\cos\left(kD\right)\right\}\left(ax\right)+\left\{2\left(1-2x^{2}\right)\left(\sin^{2}\left(kD\right)-\frac{\cos^{2}\left(kD\right)}{2x^{2}}\right)+4\sin\left(kD\right)\cos\left(kD\right)\right\}\left(1-a^{2}x^{2}/2\right)+\left\{4x\left(\sin^{2}\left(kD\right)-\frac{\cos^{2}\left(kD\right)}{2x^{2}}\right)-\frac{2\left(1-2x^{2}\right)}{x}\sin\left(kD\right)\cos\left(kD\right)\right\}\left(ax\right)=4{\left(ax^{2}+1\right)}^{2}\sin^{2}\left(kD\right)+\left(4+a^{2}\right)\cos^{2}\left(kD\right)-\left(4+4ax^{2}\right)\sin\left(kD\right)\cos\left(kD\right)$$

37
$$\frac{4{\left|M_{21}^{\prime }\right|}^{2}}{M^{2}}\approx \left(1+2x^{2}-2x\right)\left(\sin^{2}\left(kD\right)+\frac{\cos^{2}\left(kD\right)-2x\sin\left(kD\right)\cos\left(kD\right)}{2x^{2}}\right)\exp\left(ax\right)+\left(1+2x^{2}+2x\right)\left(\sin^{2}\left(kD\right)+\frac{\cos^{2}\left(kD\right)+2x\sin\left(kD\right)\cos\left(kD\right)}{2x^{2}}\right)\exp\left(-ax\right)+\left(1-2x^{2}+2ix\right)\left(\sin^{2}\left(kD\right)-\frac{\cos^{2}\left(kD\right)-2\mathrm{ix}\sin\left(kD\right)\cos\left(kD\right)}{2x^{2}}\right)\exp\left(iax\right)+\left(1-2x^{2}-2ix\right)\left(\sin^{2}\left(kD\right)-\frac{\cos^{2}\left(kD\right)+2\mathrm{ix}\sin\left(kD\right)\cos\left(kD\right)}{2x^{2}}\right)\exp\left(-iax\right)=\left\{2\left(1+2x^{2}\right)\left(\sin^{2}\left(kD\right)+\frac{\cos^{2}\left(kD\right)}{2x^{2}}\right)+4\sin\left(kD\right)\cos\left(kD\right)\right\}\cosh\left(ax\right)-\left\{4x\left(\sin^{2}\left(kD\right)+\frac{\cos^{2}\left(kD\right)}{2x^{2}}\right)+\frac{2\left(1+2x^{2}\right)}{x}\sin\left(kD\right)\cos\left(kD\right)\right\}\sinh\left(ax\right)+\left\{2\left(1-2x^{2}\right)\left(\sin^{2}\left(kD\right)-\frac{\cos^{2}\left(kD\right)}{2x^{2}}\right)-4\sin\left(kD\right)\cos\left(kD\right)\right\}\cos\left(ax\right)-\left\{4x\left(\sin^{2}\left(kD\right)-\frac{\cos^{2}\left(kD\right)}{2x^{2}}\right)+\frac{2\left(1-2x^{2}\right)}{x}\sin\left(kD\right)\cos\left(kD\right)\right\}\sin\left(ax\right)\approx 4{\left(ax^{2}-1\right)}^{2}\sin^{2}\left(kD\right)+\left(4+a^{2}\right)\cos^{2}\left(kD\right)+\left(4-4ax^{2}\right)\sin\left(kD\right)\cos\left(kD\right)$$

Note that the transmission is

38
$$T=\frac{1}{|M_{11}^{\prime }{|}^{2}}∼\frac{1}{M^{2}}\approx 0$$

Thus, finally, the absorption is

39
$$A\approx 1-R=1-\frac{4{\left(ax^{2}-1\right)}^{2}\sin^{2}\left(kD\right)+\left(4+a^{2}\right)\cos^{2}\left(kD\right)+\left(4-4ax^{2}\right)\sin\left(kD\right)\cos\left(kD\right)}{4{\left(ax^{2}+1\right)}^{2}\sin^{2}\left(kD\right)+\left(4+a^{2}\right)\cos^{2}\left(kD\right)-\left(4+4ax^{2}\right)\sin\left(kD\right)\cos\left(kD\right)}=\frac{16ax^{2}\sin^{2}\left(kD\right)-8ax^{2}\sin\left(kD\right)\cos\left(kD\right)}{4{\left(ax^{2}+1\right)}^{2}\sin^{2}\left(kD\right)+\left(4+a^{2}\right)\cos^{2}\left(kD\right)-\left(4+4ax^{2}\right)\sin\left(kD\right)\cos\left(kD\right)}$$

As shown in Figure 2, we focus on the maximal constructive interference limit: *kD* ≈ *π*/2. Hence,

40
$$A\approx \frac{4ax^{2}}{{\left(ax^{2}+1\right)}^{2}}\equiv \frac{4x^{\prime }}{{\left(x^{\prime }+1\right)}^{2}}$$

We will find the maximum by taking the derivative of **A** with respect to *x*′

41
$$\frac{\partial A}{\partial x^{\prime }}=\frac{4\left(x^{\prime }-1\right)}{{\left(x^{\prime }+1\right)}^{3}}=0$$

42
$$2\pi L_{M}\chi_{M}/\lambda_{M}=ax^{2}=x^{\prime }=1$$

Hence finally, we obtain the approximate analytical formula for the ridge in the limit of small thickness for Figure 3(b): 2*πL*_*M*_ *χ*_*M*_/*λ*_*M*_ = 1. The maximal absorption is **A**(*x*′ = 1) = 1.

## Appendix B: A Realistic Silver Mirror

In this appendix, we present the supplementary results obtained for the more realistic silver mirror; we first note that the absorption behavior is not very different from that obtained for the ideal mirror. Some interesting differences may still be seen: the fundamental interference distance is smaller than λ_*M*_/4 because a realistic silver mirror with a finite complex-valued dielectric constant *n*_*Ag*_ is penetrable, and thus we only need a smaller distance *D*_*c*_. Moreover, a realistic and penetrable silver mirror breaks the quantitative agreement between TMM and FDTD. For perfect agreement, one must also require that within the frequency window of interest, the real-valued dielectric constant must dominate over the complex-valued dielectric constant (e.g., using a dielectric mirror). After all, TMM cannot fully capture the absorption due to irreversible loss from the metallic structure and molecular intrinsic relaxation. With this limitation in mind, as shown in Figure 5(a), TMM shows an unphysical vanishing of the absorption in the case where all EM signals pass through the system and get reflected by the silver mirror; TMM completely misses all irreversible absorption effects due to penetration depth and thus cannot rigorously agree with FDTD.

## Appendix C: Parameters

In this appendix, we list all parameters in the TMM and FDTD simulations in Table 1.

**Table 1:** Parameters for Maxwell-Bloch Simulation

Name	Value
Molecules resonant frequency ℏω_*ge*_	1.9 eV
Molecules resonant wavelength λ_*M*_	652 nm
Thickness of the silver mirror	70 nm
Distance between silver mirror and molecular layer in Figure 1(c)	130 nm
Drude model for silver	Ω_*d*_ = 8.90 eV
	Γ_*d*_ = 0.243 eV
Transition dipole moment	μ_*x*_ = 5 D
Simulation grid size (1D)	2000 nm
Number of grid points	4000
Number of density matrices	20
Grid resolution (*dx*)	0.5 nm
Time step (dt)	*dx*/2/*c*_0_
CPML grid points	19
